# Supplementary material for: Acceptance and compliance with micronutrient powder (MNP) among children aged 6–23 months in northern Nigeria
Source: PLOS Glob Public Health. 2022 Oct 17;2(10):e0000961. doi: 10.1371/journal.pgph.0000961 (PMC10022258; doi:10.1371/journal.pgph.0000961)
Supplement: S1 Dataset — (ZIP) [file pgph.0000961.s008.zip › S8_Dataset/Spot Checks.zip!/Spot Checks/080715_K_J_AD_2_SC.a.pdf]

SPOT CHECK RECORDING FORM

State: Kebbi LGA: Jega Community: Rugga

Sex of index child: F   Age of index child (in months): 20 months

Spot Check Information

1. **Date of MNP distribution to household** (circle option below based on LGA name above)**:**
   - **Kebbi:** BirninKebbi&Jega LGA: **June 16**
   - **Kebbi:** Danko-wasagu: **June 17**
   - **Adamawa:**Song, Ganye: **June 18**
   - **Adamawa:**Guyuk **June 19**
2. **Date of Spot Check Observation: 8th july 2015**
3. **Number of sachets remaining**(Ask the caregiver to show you and count the specific number of sachets remaining)**:**

39

4. **Number of sachets that should remainusing one MNP sachet per day** (Analyst only)**:**

39

5. **Number of sachets remaining / number of sachets should be remaining**(Analyst only)**:**

   ______39_____ / _____39______ = _____100______ %

Probes

Question 1. You have **XX** MNP sachets remaining. Explain how easy or difficult it was to use the MNP product on a daily basis for your child 6 – 23 months? (Circle one answer)

Very Difficult **Difficult** **Easy** **Very Easy**

Question 2. Describe any specific challenges that you faced that made the MNP difficult to use on a daily basis?

I don't have any challenge with it, the only challenge is that the boy doesn't like to take kunu and I have to force him to take it

Question 3. Describe specific aspects of the MNP product that you or your child enjoyed that made it easier to use on a daily basis?

It has been easy to administer as it has increased the child's appetite

Question 4. What questions do you have about the MNP based on your experience using it?

No question

Question 5. What specific recommendations do you have that we can do as a program to make the MNP easier to use on a daily basis?

I don't have any recommendation
